# Thermo-diffusion of excited photothermal semiconductor under laser pulse and ramp heating with acoustic pressure

**DOI:** 10.1016/j.heliyon.2024.e38388

**Published:** 2024-09-24

**Authors:** Hashim M. Alshehri, Khaled Lotfy

**Affiliations:** a Mathematics Department, Faculty of Science, King Abdulaziz University, Jeddah, 21521, Saudi Arabia; b Department of Mathematics, Faculty of Science, Zagazig University, P.O. Box44519, Zagazig, Egypt; c Department of Mathematics, College of Science, Taibah University, P.O. Box 344, Al-Madinah Al-Munawarah, 30002, Saudi Arabia

**Keywords:** Diffusion, Acoustic wave, Optoelectronics, Semiconductors, Photo-thermoelasticity, Recombination processes

## Abstract

The goal of this work is to provide a novel mathematical model that explains how certain physical variables propagate (acoustic-thermal-mechanical diffusive) as waves in a photoexcited non-Gaussian laser pulse semiconductor medium. Under the impact of acoustic pressure, the isotropic and homogeneous semiconductor medium is discussed concerning the fundamental equations according to charge carrier recombination processes with optoelectronic properties. Given the impact that relaxation times have on the governing equations. Laplace transforms were utilized in a one-dimensional (1D) context to examine essential non-dimensional properties such as displacement, stress components, carrier density, temperature, and acoustic pressure in order to mathematically answer the required problem. By imposing specific initial and boundary conditions, inverse Laplace transformations were employed to generate precise solutions for the numerical modeling of different physical quantities depicted graphically. The graphical representation of wave propagation data was followed by a theoretical analysis and interpretation, emphasizing the influence of other factors (such as heat rise time, relaxation times, and laser pulse effects) on the observed occurrences.

## Nomenclature

λ,μ: Lame's constants
$n_{0}$: Electron's concentration
$T_{0}$: Absolute temperature
$\gamma =\left(3\lambda +2\mu \right)\alpha_{t}$: The thermoelastic coupling coefficient
I: The specific heat ratio (=1.666)
ρ: Medium density
$\alpha_{n}$: Electrons thermo-diffusive parameters
$t^{n}$: The electron's relaxation time.
$\alpha_{t}$: The volume expansion factor
$\tau_{q}$: The elastic relaxation time.
$\tau_{\theta }$: Thermal relaxation time.
$C_{e}$: Specific heat at a constant strain of the medium
K: The thermal conductivity of the medium
$t_{1}^{n}$: The lifetime.
$E_{g}$: The energy gap of the medium of the semiconductor
$\delta_{n}=\left(2\mu +3\lambda \right)d_{n}$: The electrons elastodiffusive parameter
$d_{n}$: The coefficients of electronic deformation
$m_{nq},m_{qn},m_{hq},m_{qh}$: Peltier-Seebeck- Dufour-Soret-like constants
$D_{n}$: The diffusion coefficients of the electrons
$a_{Qn},a_{Qh},a_{Q},a_{n},a_{h}$: The flux-like constants
$c_{s}$: The speed of sound in the material (=8430 m/s)
p: The power intensity of the laser
δ: The optical absorption coefficient
Ω: The pulse parameter
β: The volumetric thermal expansion coefficient (2.56×10−6Co)

## 1 Introduction

At the temperature of absolute zero, semiconductors have insulating properties, but they transition to a conductive state as the temperature increases. When photo-excited free carriers come into contact with them, they experience electrical or thermoelastic deformation. Doping enhances the electrical characteristics of the material to suit different applications. While insulators have higher resistance and conductors have lower resistance, the resistance reduces as the temperature increases. Semiconductors play a vital role in several technological applications, such as temperature sensors, transistors, and electrical switches. Comprehending the transmission of elastic waves in semiconductors is crucial for practical uses, as investigated by the photo-thermoelasticity hypothesis. When a semiconductor's surface is exposed to laser or sunlight, the excited electrons form plasma waves. This comprehension facilitates the utilization of semiconductors in contemporary electronics and environmentally friendly energy solutions.

Biot [1] introduced the coupled-dynamic thermoelasticity theory, which encompasses the concepts of elastic and thermal wave propagation. In contrast, Lord and Shulman (LS) [2] and Green and Lindsay (GL) [3] incorporated thermal memories into the heat equation and the equation of motion for thermoelastic bodies, resulting in novel models of thermoelasticity theory. These models, known as the generalized thermoelastic theory, resolve the paradoxes in the theory of uncoupled thermoelasticity by linking the heat equation with the governing equations for elastic materials [[4], [5], [6]]. This theory has found numerous applications including the study of interference between electromagnetic, mechanical, and thermal-elastic waves, and the investigation of the effect of magnetic fields on elastic solid objects [7,8]. Abbas et al. [9,10] examined the variable thermal conductivity of the two-dimensional generalized magneto-thermoelastic problem using a laser pulse during thermal shock. Marin et al. [11,12] introduced some new results according to the Moore‐Gibson‐Thompson for thermoelastic media and nonlinear bioheat model in the context of the dipolar bodies. On the other hand, Abbas [13] used the functional graded thermoelastic material to study the fractional order heat. Carrera et al. [14] Investigated the vibrational analysis according to the two-temperature theory for an axially moving microbeam. Abbas et al. [[15], [16], [17]] studied the response of thermal source according to mass diffusion and thermoelastic interaction in a transversely isotropic half-space subjected to a moving heat source and fractional Order.

Maruszewski [18,19] has introduced new theoretical models that explore the interplay of elastic, thermal, and charge carrier fields within elastic semiconductors. Recent research [20,21] has delved into the thermal diffusivity of semiconductors during heat and mass transfer processes, considering the overlap between thermal, elastic, and plasma waves according to meshless method. Contemporary investigations into sensitive photoacoustic phenomena in semiconductor materials utilize the photothermal (PT) approach [22]. This PT mechanism, used to assess the physical properties of semiconductors, relies on both TED and ED deformations. The response of semiconductor mediums to laser beams, electromagnetic radiation, and acoustic waves has been under scrutiny [23]. A multitude of researchers [[24], [25], [26]] have tackled various aspects of elastic semiconductor materials within the framework of photo-thermoelasticity theory. The alteration of thermal conductivity during PT transport operations has been explored in terms of the absorbed optical energy on the free surfaces of both homogeneous and non-homogeneous semiconductor materials [[27], [28], [29], [30], [31]]. In the study of semiconductors within PT theory, the interaction between electrons and holes is disregarded in all aforementioned experiments. However, the impact of two separate temperatures is dismissed in the absence of a heat source. On the contrary, Sarkar et al. [32,33] have analyzed the propagation of photo-thermal waves in nonlocal semiconductors with memory-dependent derivatives using the two-temperature theory, exploring a range of generalized photo-thermoelasticity theories.

Sound waves are generated when mechanical vibrations propagate through substances or the air [34]. They play a crucial role in the fields of physics, engineering, and medicine. The study of semiconductor materials, particularly in relation to photoacoustic phenomena, heavily relies on the analysis of acoustic pressure. For example, the manipulation of sound waves can be employed to investigate the arrangement of energy levels and the movement of electrons in semiconductors, providing insights on their electronic band structure and electron mobility [35]. Additionally, the response of semiconductors to acoustic waves can be utilized in various applications, such as the development of ultrasonic sensors and transducers, as well as in the field of photoacoustic imaging [36]. Understanding the effect of acoustic pressure on semiconductors is crucial for optimizing their performance in electronic devices and for advancing research in materials science [37]. Lotfy et al. [38] introduced a novel model that describes the photoacoustic and plasmaelastic phenomena of a nanoscale semiconductor medium heated by laser pulsed. The creation of acoustic (or elastic) waves through the transient thermal heating of a material is emerging as a potent technique for characterizing the material and examining its microstructure.

In previous studies examining the effect of electron diffusion in semiconductors, the effect of acoustic pressure was not taken into account. The primary objective of this study is to examine the influence of electron diffusion in the presence of acoustic pressure. The investigation utilizes a homogeneous, isotropic semiconductor medium during photo-generated excitation to investigate the propagation of elasto-thermodiffusive waves under the impact of a laser pulse (as an internal heat source). A novel mathematical model for photo-acoustic-thermoelasticity, presented in dimensionless form, is developed. Analytical Laplace transforms in one dimension (1D) are applied to the governing partial differential equation under appropriate conditions. The Laplace transform inversion numerically is used to obtain complete solutions for key quantities such as acoustic pressure, thermal, mechanical, holes, and carrier density distributions. These solutions are then graphically presented to allow for analytical validation. The study also explores the thermal memory of silicon media and the effects of laser pulse parameters, with numerical results presented graphically and theoretical analysis.

## 2 Basic equations

Let's consider a 1D Cartesian coordinate system for the deformation of an initially at a reference temperature $T_{0}$, elastic, homogeneous, isotropic, and thermally conducting n-type semiconductor rod. In this scenario, all field values are assumed to be independent of the y and z coordinates. As per the semiconductor's electric neutrality, the governing field equations for temperature variation T(x,t), displacement u(x,t), acoustic pressure P(x,t), and carrier density (electron diffusion field) N(x,t) in the linear theory of photo-thermoelasticity for semiconductors are dependent on time *t* and the x-axis. The main equations in the context of the heat source impact (the non-Gaussian laser pulses effect) according to the charge carrier diffusion, can be written without body forces and electromagnetic pressure [[18], [19], [20], [21], [22], [23]]:

(1) $$\begin{array}{l} K\left(1+\tau_{\theta }\frac{\partial }{\partial t}\right)\frac{\partial^{2}T}{\partial x^{2}}+m_{nq}\frac{\partial^{2}N}{\partial x^{2}}-\rho a_{1}^{n}\frac{\partial N}{\partial t}-\frac{\rho a_{1}^{n}}{t^{n}}N-\\ \left(1+\tau_{q}\frac{\partial }{\partial t}\right)\left[\rho \;C_{e}\frac{\partial T}{\partial t}+\rho \;T_{0}\alpha_{n}\frac{\partial N}{\partial t}+T_{0}\gamma \frac{\partial }{\partial x}\frac{\partial u}{\partial t}\right]=\left(1+\tau_{q}\frac{\partial }{\partial t}\right)p\delta e^{-\left(\Omega t+\delta x\right)} \end{array}\text{,}$$

(2) $$m_{qn}\frac{\partial^{2}T}{\partial x^{2}}+D_{n}\rho \frac{\partial^{2}N}{\partial x^{2}}-\rho \left(1-a_{2}^{n}\;T_{0}\alpha_{n}+t^{n}\frac{\partial }{\partial t}\right)\frac{\partial N}{\partial t}-a_{2}^{n}\left[\rho \;C_{e}\frac{\partial T}{\partial t}+T_{0}\gamma \frac{\partial }{\partial x}\frac{\partial u}{\partial t}\right]=-\frac{\rho }{t_{1}^{n}}\left(1+t^{n}\frac{\partial }{\partial t}\right)N\text{,}$$

(3) $$\rho \;\frac{\partial^{2}u}{\partial t^{2}}=\left(2\mu +\lambda \;\right)\frac{\partial^{2}u}{\partial x^{2}}-\gamma \left(1+\tau_{\theta }\frac{\partial }{\partial t}\right)\frac{\partial T}{\partial x}-\delta_{n}\frac{\partial N}{\partial x}\text{,}$$

where $a_{1}^{n}=\frac{a_{Qn}}{a_{Q}}$, $a_{1}^{h}=\frac{a_{Qh}}{a_{Q}}$, $a_{2}^{n}=\frac{a_{Qn}}{a_{n}}$, $a_{2}^{h}=\frac{a_{Qh}}{a_{h}}$.

The main characteristics of photo-thermoelasticity theories are determined by the temperature gradient phase-lag parameters $\tau_{\theta }$ and $,\tau_{q}$. The dual phase-lag (DPL) model is introduced when $0\leq \tau_{\theta }<\tau_{q}$. Conversely, when $\tau_{\theta }=0$, the Lord and Shulman (LS) model is derived. At $\tau_{\theta }=\tau_{q}=0.0$, the model represents the coupled thermoelasticity (CT) theory.

The phenomenon of generating acoustic waves (with initial acoustic pressure $P_{0}$) due to the thermal activity of plasma (carrier density diffusion) within a semiconductor medium is referred to as the photoacoustic effect as [[37], [38], [39]]:

(4) $$\frac{\partial^{2}P\left(x,t\right)}{\partial x^{2}}-\frac{1}{c_{s}^{2}}\frac{\partial^{2}P\left(x,t\right)}{\partial t^{2}}+I\beta \frac{\partial^{2}T\left(x,t\right)}{\partial t^{2}}=0\text{.}$$

The constitutive equation in 1D when the impact of the electron-thermal fields is taken into account is [29,30]:

(5) $$\sigma_{xx}=\left(2\mu +\lambda \right)\frac{\partial u}{\partial x}-\left(\gamma \left(1+\tau_{\theta }\frac{\partial }{\partial t}\right)T+\delta_{n}N\right)=\sigma \text{.}$$

For more simplicity, the following quantities can be introduced [31,39]:

(6) $$\begin{array}{l} \left(x^{\prime },u^{\prime }\right)=\frac{\omega^{*}\left(x,u\right)}{C_{T}},\left(t^{\prime },\tau_{q}^{\prime },\tau_{\theta }^{\prime },{t^{n}}^{\prime },{t_{1}^{n}}^{\prime }\right)=\omega^{*}\left(t,\tau_{q},\tau_{\theta },t^{n},t_{1}^{n}\right),\Omega^{\prime }=\frac{\Omega K}{\rho C_{w}C_{T}^{2}}\text{,}\\ \beta^{2}=\frac{C_{T}^{2}}{C_{L}^{2}},k=\frac{K}{\rho C_{e}},\sigma_{ij}^{\prime }=\frac{\sigma_{ij}}{2\mu +\lambda },N^{\prime }=\frac{\delta_{n}\left(N\right)}{2\mu +\lambda },C_{T}^{2}=\frac{2\mu +\lambda }{\rho },C_{L}^{2}=\frac{\mu }{\rho }\text{,}\\ \omega^{*}=\frac{C_{e}\left(\lambda +2\mu \right)}{K},\left({\bar{\delta }}_{n},{\bar{\delta }}_{h}\right)=\frac{\left(\delta_{n}n_{0},\delta_{h}h_{0}\right)}{\gamma T_{0}},\delta^{\prime }=\frac{\delta K}{\rho C_{w}C_{T}},T^{\prime }=\frac{\gamma T}{2\mu +\lambda },P^{\prime }=\frac{P}{P_{0}}\text{.} \end{array}$$

If the primes are disregarded for convenience, the basic equations can be written as follows:

(7) $$\begin{array}{l} \left(1+\tau_{\theta }\frac{\partial }{\partial t}\right)\frac{\partial^{2}T}{\partial x^{2}}+\left\{\alpha_{1}\frac{\partial^{2}}{\partial x^{2}}-\alpha_{2}\left(1+\tau_{q}\frac{\partial }{\partial t}\right)\frac{\partial }{\partial t}-\alpha_{3}\frac{\partial }{\partial t}-\alpha_{4}\right\}N-\\ \left(1+\tau_{q}\frac{\partial }{\partial t}\right)\frac{\partial T}{\partial t}-\left(1+\tau_{q}\frac{\partial }{\partial t}\right)\varepsilon_{1}\frac{\partial }{\partial t}\left(\frac{\partial u}{\partial x}\right)=\left(1+\tau_{q}\frac{\partial }{\partial t}\right)\Gamma_{1}e^{-\left(\Omega t+\delta x\right)} \end{array}\text{,}$$

(8) $$\left\{\frac{\partial^{2}}{\partial x^{2}}-\alpha_{8}\frac{\partial }{\partial t}\right\}T+\left\{\alpha_{9}\frac{\partial^{2}}{\partial x^{2}}-\left(\alpha_{10}+t^{n}\frac{\partial }{\partial t}\right)\frac{\partial }{\partial t}\alpha_{11}+\left(1+t^{n}\frac{\partial }{\partial t}\right)\frac{\alpha_{11}}{t^{n}}\right\}N-\alpha_{13}\;\frac{\partial }{\partial x}\frac{\partial u}{\partial t}=0\text{,}$$

(9) $$\left\{\frac{\partial^{2}}{\partial x^{2}}-\frac{\partial^{2}}{\partial t^{2}}\right\}u-\left(1+\tau_{\theta }\frac{\partial }{\partial t}\right)\frac{\partial T}{\partial x}-\frac{\partial N}{\partial x}=0\text{,}$$

(10) $$\left(\frac{\partial^{2}}{\partial x^{2}}-\varepsilon_{p}\frac{\partial^{2}}{\partial t^{2}}\right)P-\eta_{p}\frac{\partial^{2}T}{\partial t^{2}}=0\text{,}$$

where

$$\begin{array}{l} \alpha_{1}=\frac{m_{nq}\alpha_{t}}{d_{n}\;K},\alpha_{2}=\frac{T_{0}\alpha_{n}}{C_{e}},\alpha_{3}=\frac{a_{1}^{n}}{C_{e}},\alpha_{4}=\frac{a_{1}^{n}\gamma }{C_{e}\tau^{n}\left(2\mu +\lambda \right)},\alpha_{5}=\frac{\gamma m_{hq}\;h_{0}}{\left(2\mu +\lambda \right)K},\varepsilon_{p}=\frac{C_{T}^{2}}{c_{s}^{2}}\text{,}\\ \alpha_{6}=\frac{T_{0}\;\alpha_{h}\;K\;h_{0}}{C_{e}},\alpha_{7}=\frac{a_{1}^{h}\gamma \omega^{*}}{t^{h}K},\alpha_{8}=\frac{a_{2}^{n}K}{m_{qn}},\alpha_{9}=\frac{D_{n}\rho \alpha_{t}}{m_{qn}\;d_{n}},\alpha_{10}=1-a_{2}^{n}T_{0}\alpha_{n},\eta_{p}=-\partial \beta \frac{C_{T}^{2}}{\gamma P_{0}C_{e}}\text{,}\\ \alpha_{11}=\frac{\alpha_{t}K}{m_{qn}\;d_{n}C_{e}},\varepsilon_{1}=\frac{T_{0}\gamma^{2}\omega^{*}}{\rho K},a^{*}=\frac{\omega^{{*}^{2}}a}{C_{T}^{2}},\alpha_{13}=\frac{a_{2}^{n}\gamma^{2}T_{0}\omega^{*}}{\rho \;m_{qn}},\Gamma_{1}=\frac{p\delta \left(1-\tau_{q}\Omega \right)}{\rho C_{w}C_{T}}\text{.} \end{array}$$

To solve the problem mathematically, the following non-dimensional initial conditions can be introduced (this can be tackled during the Laplace transform strategy):

$${u\left(x,t\right)|}_{t=0}={\frac{\partial u\left(x,t\right)}{\partial t}|}_{t=0}=0,{T\left(x,t\right)|}_{t=0}={\frac{\partial T\left(x,t\right)}{\partial t}|}_{t=0}=0,{N\left(x,t\right)|}_{t=0}={\frac{\partial N\left(x,t\right)}{\partial t}|}_{t=0}=0\text{,}$$

(11) $${P\left(x,t\right)|}_{t=0}={\frac{\partial P\left(x,t\right)}{\partial t}|}_{t=0}=0\text{.}$$

## 3 The mathematical solutions

Partial differential equations can be effectively solved with the Laplace transform technique. Nevertheless, the transformation domain solutions' formulations are typically complex and cannot be analytically inverted to the physical domain. The Laplace transform is employed in the time-space domain to convert the partial differential equations into ordinary differential equations based on the given initial conditions. The function Δ(x,t) can be derived using the Laplace transform in the following manner:

(12) $$L\left(\Delta \left(x,t\right)\right)=\bar{\Delta }\left(x,s\right)=\int_{0}^{\infty }\Delta \left(x,t\right)\exp\left(-st\right)\;d\;t\text{.}$$

Equation (12) of the Laplace transform can be applied to derive the following subsequent equations:

(13) $$\left(q_{1}\;D^{2}-q_{2}\right)\bar{T}+\left(\alpha_{1}D^{2}-q_{3}\right)\bar{N}-q_{5}D\bar{u}=\Gamma_{2}e^{-\delta x}\text{,}$$

(14) $$\left(D^{2}-q_{7}\right)\bar{T}+\left(\alpha_{9}D^{2}-q_{6}\right)\bar{N}-q_{9}D\bar{u}=0\text{,}$$

(15) $$\left(D^{2}-s^{2}\right)\bar{u}-q_{14}D\bar{T}-D\bar{N}=0\text{,}$$

(16) $$\left(D^{2}-\Theta_{1}\right)\bar{P}-\Theta_{2}\bar{T}=0\text{,}$$

(17) $$\bar{\sigma }=\alpha_{23}\left(D\bar{u}-\left(\;\left(1+s\tau_{\theta }\right)\bar{T}+\bar{N}\right)\right)\text{,}$$

where $D=\frac{d}{dx}$, $q_{1}=\left(1+\tau_{\theta }s\right)$, $\beta =\frac{1}{a^{*}}$, $q_{2}=\left(1+\tau_{q}s\right)\;s$, $q_{3}=\left(\alpha_{2}\left(1+\tau_{q}s\right)s+\alpha_{3}s+\alpha_{4}\right),\begin{array}{l} q_{4}=\left(1+\tau_{q}s\right)\alpha_{6}+\alpha_{7},q_{5}=\left(1+\tau_{q}\;s\right)\varepsilon_{1}\;s,q_{6}=\left(\alpha_{10}+t^{n}s\right)s\alpha_{11}-\left(1+t^{n}s\right)\frac{\alpha_{11}}{t^{n}},\Theta_{1}=\eta_{p}s^{2}\text{,}\\ q_{7}=\alpha_{8}\;s,q_{8}=\alpha_{12}s,q_{9}=\alpha_{13}\;s,q_{14}=\left(1+\tau_{\theta }s\right),\Gamma_{2}=\frac{\Gamma_{1}\left(1+\tau_{q}s\right)}{s+\Omega },\Theta_{2}=\varepsilon_{p}s^{2}\text{.} \end{array}$

Applying the method of elimination between variables $\bar{T},\bar{u},\bar{N}$ and $\bar{P}$ in the preceding equations yields the following differential equation:

(18) $$\begin{array}{l} \left(D^{8}-\Delta_{1}D^{6}+\Delta_{2}D^{4}-\Delta_{3}D^{2}+\Delta_{4}\right)\bar{f}\left(x,s\right)=\Pi e^{-\delta x}\\ \bar{f}\left(x,s\right)=\left\{\bar{P},\bar{T},\bar{N},\bar{u}\right\} \end{array}\}\text{,}$$

where

$$\Pi =\frac{\Gamma_{1}\Theta_{2}\left(\alpha_{9}\delta^{4}-\Theta_{5}\delta^{2}+\Theta_{6}\right)}{\alpha_{9}q_{1}-\alpha_{1}}\text{,}$$

$$\begin{array}{l} \Delta_{1}=\frac{1}{\alpha_{9}q_{1}-\alpha_{1}}\left\{\alpha_{9}\left(\Theta_{1}q_{1}+q_{2}\right)-\alpha_{1}\left(\Theta_{1}+\Theta_{8}\right)+q_{1}\Theta_{5}-q_{3}+q_{5}\Theta_{3}\right\},\Theta_{9}=q_{7}s^{2}\text{,}\\ \Delta_{2}=\frac{1}{\alpha_{9}q_{1}-\alpha_{1}}\left\{\Theta_{5}\left(\Theta_{1}q_{1}+q_{2}\right)+\Theta_{1}\left(\alpha_{9}q_{2}+q_{3}\right)+q_{1}\Theta_{6}+\Theta_{9}\alpha_{1}+\Theta_{8}\left(\Theta_{1}\alpha_{1}+q_{3}\right)+q_{5}\left(\Theta_{1}\Theta_{3}+\Theta_{4}\right)\right\}\text{,}\\ \Delta_{3}=\frac{-1}{\alpha_{9}q_{1}-\alpha_{1}}\left\{\Theta_{1}\left(\Theta_{5}q_{1}+\Theta_{8}q_{3}\right)+\Theta_{6}\left(\Theta_{1}q_{1}+q_{2}\right)+\Theta_{9}\left(\Theta_{1}\alpha_{1}+q_{3}\right)\right\},\Theta_{8}=q_{7}+q_{9}q_{14}+s^{2}\text{,}\\ \Delta_{4}=\frac{\Theta_{1}\left(\Theta_{6}q_{2}+\Theta_{9}q_{3}-\Theta_{4}q_{5}\right)}{\alpha_{9}q_{1}-\alpha_{1}},\Theta_{3}=1-\alpha_{9}q_{14},\Theta_{4}=q_{7}-q_{6}q_{14},\Theta_{5}=q_{6}+q_{9}+s^{2}\alpha_{9},\Theta_{6}=q_{6}s^{2}\text{.} \end{array}$$

By displaying the differential characteristic equation in the manner shown below:

(19) $$\left(D^{2}-m_{1}^{2}\right)\left(D^{2}-m_{2}^{2}\right)\;\left(D^{2}-m_{3}^{2}\right)\left(D^{2}-m_{4}^{2}\right)\bar{f}\left(x,s\right)=\Pi e^{-\delta x}\text{.}$$

In the above equation, $m_{i}\left(i=1,2,3,4\right)$ denote the roots of equation (19) which can be chosen as a real (when x→∞).

The general linear solutions for the acoustic pressure obtained in equation (18) are illustrated as follows:

(20) $$\bar{P}\left(x,s\right)=\sum_{i=1}^{4}\Lambda_{i}\left(s\right)\;e^{-k_{i}x}+\Psi e^{-\delta x}\text{,}$$

where $\Psi =1/\left(\delta^{8}-\Delta_{1}\delta^{6}+\Delta_{2}\delta^{4}-\Delta_{3}\delta^{2}+\Delta_{4}\right)$ and $\Lambda_{i}$ are unknown parameters depend on the Laplace parameter s. Conversely, the mathematical approach can be utilized to depict the linear solutions for other essential physical quantities:

(21) $$\bar{N}\left(x,s\right)=\sum_{i=1}^{4}H_{1i}\Lambda_{i}\left(s\right)\;e^{-m_{i}x}+f_{1}\left(s\right)e^{-\delta x}\text{,}$$

(22) $$\bar{u}\left(x,s\right)=\sum_{i=1}^{4}H_{2i}\;\Lambda_{i}\left(s\right)\;e^{-m_{i}x}+f_{2}\left(s\right)e^{-\delta x}\text{,}$$

(23) $$\bar{T}\;\left(x,s\right)=\sum_{i=1}^{4}H_{3i}\;\Lambda_{i}\left(s\right)\;e^{-m_{i}x}+f_{3}\left(s\right)e^{-\delta x}\text{,}$$

(24) $$\bar{\sigma }\;\left(x,s\right)=\sum_{i=1}^{4}H_{4i}\;\Lambda_{i}\left(s\right)\;e^{-m_{i}x}+f_{4}\left(s\right)e^{-\delta x}\text{,}$$

where

$$f_{1}=-\frac{\left(\delta^{4}-\Theta_{8}\delta^{4}+\Theta_{9}\right)\left(\delta^{4}-\Theta_{1}\right)}{\Theta_{2}\left(\delta^{4}\alpha_{9}-\Theta_{5}\delta^{4}+\Theta_{6}\right)},f_{2}=-\frac{\delta \left(\Theta_{3}\delta^{2}-\Theta_{4}\right)\left(\delta^{2}-\Theta_{1}\right)}{\Theta_{2}\left(\delta^{4}\alpha_{9}-\Theta_{5}\delta^{2}+\Theta_{6}\right)}\text{,}$$

$$f_{3}=\frac{\left(\delta^{2}-\Theta_{1}\right)}{\Theta_{2}},f_{4}=-\alpha_{5}\left(\delta \;H_{2i}+\left(\left(\;1+s\tau_{\theta }\right)H_{3i}+H_{1i}\right)\right)\text{,}$$

$$H_{1i}=-\frac{\left({m_{i}}^{4}-\Theta_{8}{m_{i}}^{2}+\Theta_{9}\right)\left({m_{i}}^{2}-\Theta_{1}\right)}{\Theta_{2}\left({m_{i}}^{4}\alpha_{9}-\Theta_{5}{m_{i}}^{2}+\Theta_{6}\right)},H_{2i}=-\frac{m_{i}\left(\Theta_{3}{m_{i}}^{2}-\Theta_{4}\right)\left({m_{i}}^{2}-\Theta_{1}\right)}{\Theta_{2}\left({m_{i}}^{4}\alpha_{9}-\Theta_{5}{m_{i}}^{2}+\Theta_{6}\right)}\text{,}$$

$$H_{3i}=\frac{\left({m_{i}}^{2}-\Theta_{1}\right)}{\Theta_{2}},H_{4i}=-\alpha_{5}\left(m_{i}\;H_{2i}+\left(\left(\;1+s\tau_{\theta }\right)H_{3i}+H_{1i}\right)\right)\text{.}$$

## 4 Boundary conditions

Once the values of the unknown parameters are established, complete solutions for the relevant physical fields can be obtained. Consequently, certain thermal shifts and mechanical stresses are exerted at the interface of the medium during plasma recombination processes, affecting carrier density, electronic diffusion, and electron concentration [40,41].

(I) When x=0, the thermal gradient boundary condition depends on the time t which can be chosen as ramp type heating according to harmonically varying as:

(25) $$T=\left\{ \begin{array}{l} 0,t\leq 0\\ T_{1}\frac{t}{t_{0}},0<t<t_{0}\\ T_{1},t>t_{0} \end{array}\right.\text{.}$$

Considering the medium is in an initial state of rest at a reference temperature $T_{0}$ which rises suddenly, depending on the passage of time, to a gradual temperature of $T_{1}\frac{t}{t_{0}}$, $t_{0}$ indicates the length of time to rise the heat. When $t=t_{0}$, the constant temperature is maintained for $T_{1}$. Applying Laplace transform on the thermal ramp type conditions (25), the non-dimensional thermal condition in terms of transformed variables as:

(26) $$\sum_{i=1}^{4}H_{3i}\;\Lambda_{i}\left(s\right)+f_{3}\left(s\right)=\frac{T_{1}\left(1-e^{-st_{0}}\right)}{t_{0}s^{2}}\text{.}$$

(II) The Laplace transform is used, the mechanical condition can be chosen for normal stress as traction-free at x=0:

(27) $$\bar{\sigma }\left(0,s\right)=0\Rightarrow \sum_{i=1}^{4}H_{4i}\;\Lambda_{i}\left(s\right)+f_{4}\left(s\right)=0\text{.}$$

(III) Recombination processes arise during electron excitation and transport activities due to the thermal influence of laser pulse and ramp-type heating at x=0. In this case, the carrier density diffusivity is employed to deduce the plasma condition via Laplace transform, depicted as follows:

(28) $$\bar{N}\left(0,s\right)=\frac{ƛn_{0}}{D_{e}}\bar{R}\left(s\right)\Rightarrow \Rightarrow \sum_{i=1}^{4}H_{1i}\Lambda_{i}\left(s\right)+f_{1}\left(s\right)=\frac{ƛn_{0}}{sD_{e}}\text{.}$$

(IV) Taking into account that there is acoustic pressure on the surface x=0 at the beginning of a constant magnitude $P_{0}$, which is expressed as follows:

(29) $$\bar{P}\left(0,s\right)=\frac{P_{0}}{s}\Rightarrow \Rightarrow \sum_{i=1}^{4}\Lambda_{i}\left(s\right)+\Psi =\frac{P_{0}}{s}\text{.}$$

In the above equations, R(s) denotes the Heaviside unit step function, ƛ refers to an arbitrary parameter.

## 5 Inversion of laplace transform

The problem in the transformed domain has been completely solved. However, obtaining the inverse transform analytically in the time domain is quite challenging due to the complexity of the formulas in Equations (20), (21), (22), (23), (24). Therefore, to determine the effects on temperature, displacement, acoustic pressure, carrier density, and normal stress in real-time, we will use the numerical inverse Laplace transform technique. In the physical domain, we can use the Riemann-sum approximation method to generate numerical results. By employing the well-known equation, every function $\bar{\Phi }\left(x,s\right)$ in the Laplace transform space is converted into a physical domain $\Phi \left(x,t^{\prime }\right)$ using this approach [42,43]:

(30) $$\Phi \left(x,t^{\prime }\right)=\frac{e^{nt^{\prime }}}{t^{\prime }}\left[\frac{1}{2}Re\left(\bar{\Phi }\left(x,n\right)\right)+Re\sum_{k=0}^{N}\bar{\Phi }\left(x,n+\frac{ik\pi }{t^{\prime }}\right){\left(-1\right)}^{k}\right]\text{,}$$

where Re refers to the function real part, $i=\sqrt{-1}$ and N is largely taken freely quantity when n $\approx 4.7/t^{\prime }$ [43].

## 6 Numerical results and discussions

Numerical simulations with wave propagation graphs for the essential physical fields were used to verify this study's theoretical and analytical findings and compare them to previous studies. Following a thermal ramp and laser pulses, these graphs represented temperature (thermal distribution), acoustic pressure distribution, carrier density (plasma distribution or electron diffusion), and normal stress (mechanical or elastic wave distribution) over a short period t=0.02sec. MATLAB (2022a) plotted the principal wave distribution for silicon (Si) semiconductor material using SI units [44]. Table 1 lists Si's physical constants [[45], [46], [47], [48]]:

**Table (1):** The physical constants of Si medium.

Unit	Symbol	Value
$N/m^{2}$	λ	$6.4\times 10^{10}$
	μ	$6.5\times 10^{10}$
$\text{kg}/m^{3}$	ρ	2330
K	$T_{0}$	800
sec(s)	τ	$5x\;10^{-5}$
$K^{-1}$	$\alpha_{t}$	$4.14x\;10^{-6}$
${\text{Wm}}^{-1}K^{-1}$	k	150
J/(kgK)	$C_{e}$	695
m/s	$\tilde{s}$	2
H/m	$\mu_{0}$	$4\pi \times 10^{-7}$
${\text{vk}}^{-1}$	$m_{qn}$	$1.4\times 10^{-5}$
	$m_{nq}$	$1.4\times 10^{-5}$
	$m_{qh}$	$-0.004\times 10^{-6}$
	$m_{hq}$	$-0.004\times 10^{-6}$
$J.m^{-2}$	p	$10^{11}$
$m^{2}s^{-1}$	$D_{n}$	$0.35\times 10^{-2}$
$m^{2}s^{-1}$	$D_{h}$	$0.125\times 10^{-2}$
$m^{2}s^{-1}$	$\alpha_{n}$	$1\times 10^{-2}$
$m^{2}s^{-1}$	$\alpha_{h}$	$5\times 10^{-3}$
ps	$t_{0}$	4
$m^{-1}$	δ	3
The pulse parameter	Ω	6
K	$T_{1}$	500
s	$\tau_{q}$	0.0002
s	$\tau_{\theta }$	0.0001

### 6.1 Ramp-type heating parameter impact

**Fig. 1:**
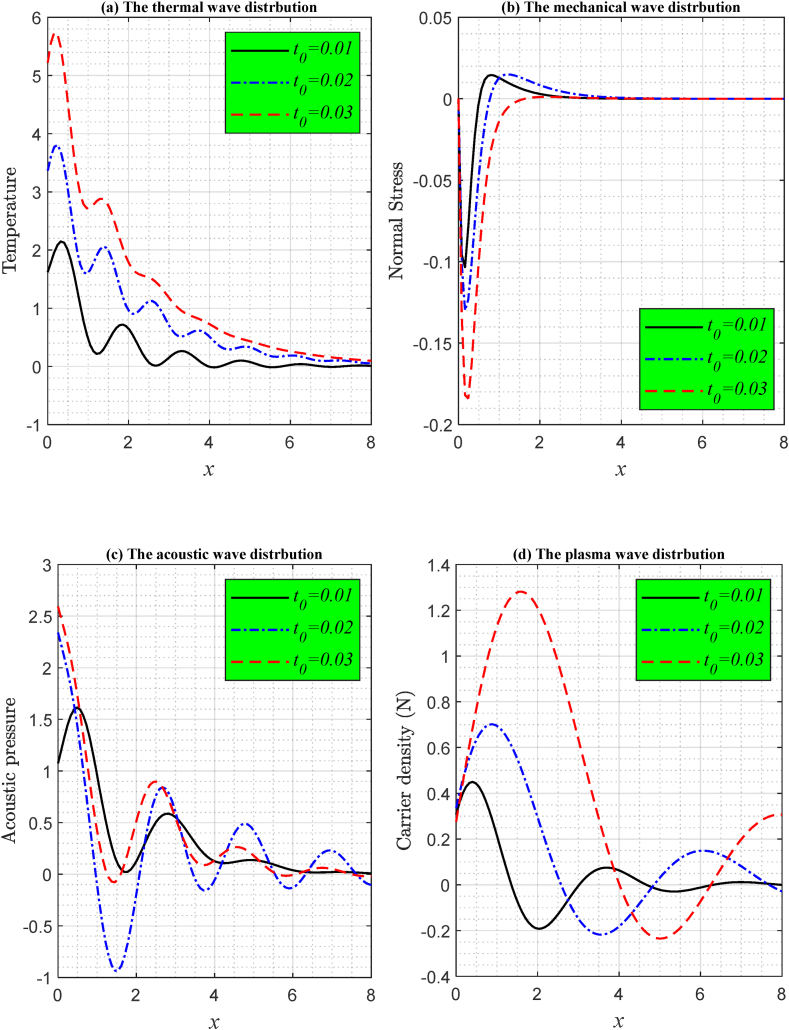
The influence of ramp-type heating parameter on the fundamental physical field distributions agonist the distance according to the LS theory with laser pulses effects.

Three different ramp-type heating parameters were selected, and the duration was set at t=0.002s to illustrate the wave propagation of temperature, stress (mechanical), acoustic pressure, and carrier charge according to LS model under the effect of the laser pulse as demonstrated in Fig. 1 ((a), (b), (c), (d)). The first subfigure (Fig. 1(a)) illustrates the impact of the ramp-type heating parameter $t_{0}$ (the length of time to rise the heat) on the temperature distribution (thermal wave) along the x-axis of the semiconductor medium. Different scenarios of the parameter were examined ($t<t_{0}$, $t=t_{0}$, and $t>t_{0}$). According to the LS model, at position x=0 (on the surface), it is observed that when $t\geq t_{0}$, the thermal wave starts at maximum value due to the ramp heating effect and laser pulse (which stems from the definition of the ramp-type heating function). With time, the distribution of temperature begins to decrease and as one goes deeper into the semiconducting material, it reaches the zero line, which is the state of physical equilibrium. In the context of photo-thermoelasticity theory, the oscillatory temperature response in a medium under ramp-type loading conditions can be explained by the interplay between thermal, mechanical, and optical fields. When a ramp-type load is applied, it induces a gradual increase in temperature, which then propagates through the material. This thermal wave interacts with the elastic and optical properties of the medium, leading to coupled thermoelastic waves. Additionally, the finite speed of wave propagation and thermal memory effects contribute to this oscillation. The material's response isn't instantaneous, and as the thermal waves move, they interact with previous waves, leading to constructive and destructive interference, which manifests as an oscillatory temperature pattern. These effects are more pronounced in materials with significant photo-thermoelastic coupling, where thermal and mechanical responses are strongly interconnected. In the second subfigure (Fig. 1(b)), the effect of the ramp-type heating parameter $t_{0}$ on the mechanical (stress) distribution under the influence of laser pulses is shown. As the stress distribution starts from zero, fulfilling the mechanical condition for freeloading. It is clear that the effect of changing $t_{0}$ appears clearly on the stress component, as the value of the stress distribution increases with the increase of the ramp-type heating coefficient $t_{0}$. From the figure it is also clear that the stress decreases sharply near the surface to reach the minimum value before it increases rapidly until it reaches equilibrium by conforming to the zero line with increasing distance. The third subfigure (Fig. 1(c)) illustrates the impact of the ramp-type heating parameter $t_{0}$ on the acoustic pressure distribution along the *x*-axis of the Si semiconductor material, as per LS theory under the influence of laser pulses. It has been demonstrated that reduced values of $t_{0}$ result in the decreased amplitude of acoustic pressure wave propagation. At the surface, the acoustic condition is met, following which the wave propagation exhibits an oscillatory pattern along the *x* axis until it stabilizes. The fourth subfigure (Fig. 1(d) shows the effect of the ramp-type heating parameter on the carrier density distribution. A higher ramp-type heating parameter $t_{0}$ typically leads to a more pronounced carrier density distribution, whereas a lower parameter value results in a less pronounced distribution. This effect can be observed when examining the carrier density against distance x. A larger ramp-type heating parameter distributes carrier density equally across the distance, while a lower parameter concentrates it. As the ramp-type heating function steadily raises the temperature, the carrier density also changes. Thus, the ramp-type heating parameter is critical to plasma wave propagation carrier density distribution.

### 6.2 The photo-thermoelasticity models

**Fig. 2:**
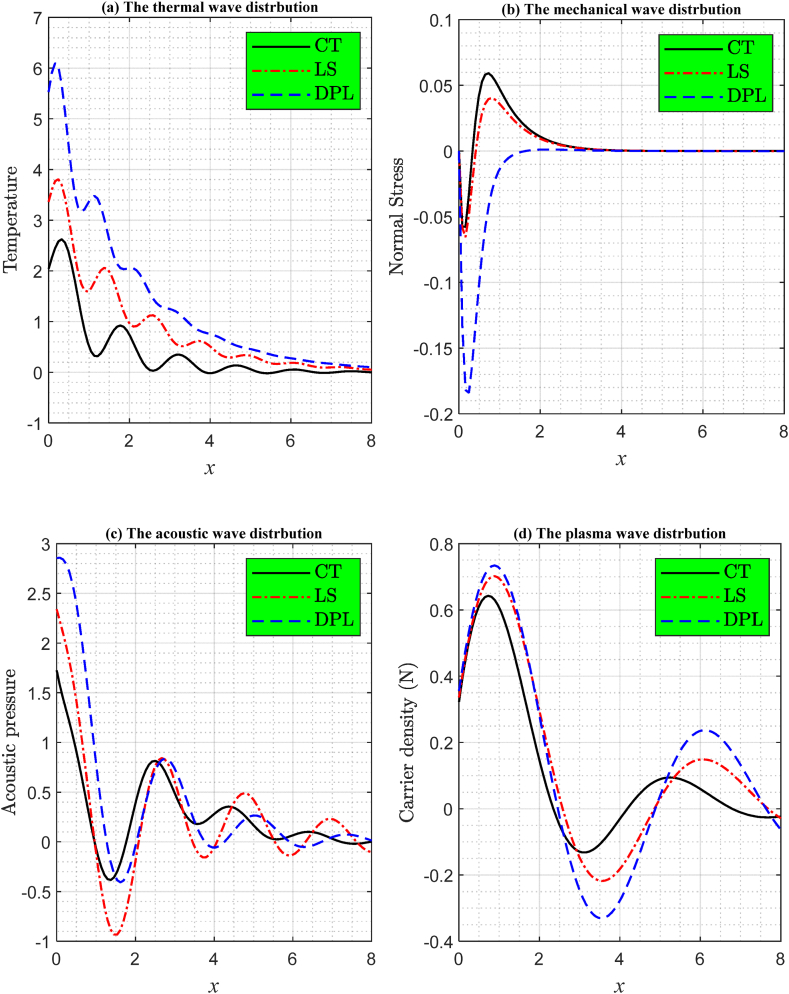
The two-dimensional graph of the main physical fields according to the various photo-thermoelasticity theories under the effect of laser pulses with ramp-type heating parameter at $t_{0}=0.02$.

Fig. 2((a), (b), (c), (d)) depict the alterations in the distribution of fundamental physical parameters across the Si semiconductor medium, influenced by various relaxation times (thermal memories) in models that adhere to the principles of photo-thermoelasticity. These figures present numerical computations of the actual dimensional parameters when subjected to extremely brief durations when the ramp-type heating parameter $t_{0}=0.02$ under the influence of laser pulses. The solid line corresponds to the coupled thermoelasticity theory (CT at $\tau_{\theta }=\tau_{q}=0.0$), the dashed line represents the dual-phase-lag (DPL, $0\leq \tau_{\theta }<\tau_{q}$) model, and the dashed-dotted line signifies the Lord and Shulman (LS, $\tau_{\theta }=0$, $0<\tau_{q}=0.0002$)) model. It is clear from Fig. 2 that relaxation times greatly affect the diffusion of wave propagation for the physical quantities under study. In addition, the numerical simulation (theoretical results in this work) of main field distributions is consistent with the experimental results, especially those related to thermal distribution and plasma distribution [49,50].

### 6.3 The laser pulses effect

**Fig. 3:**
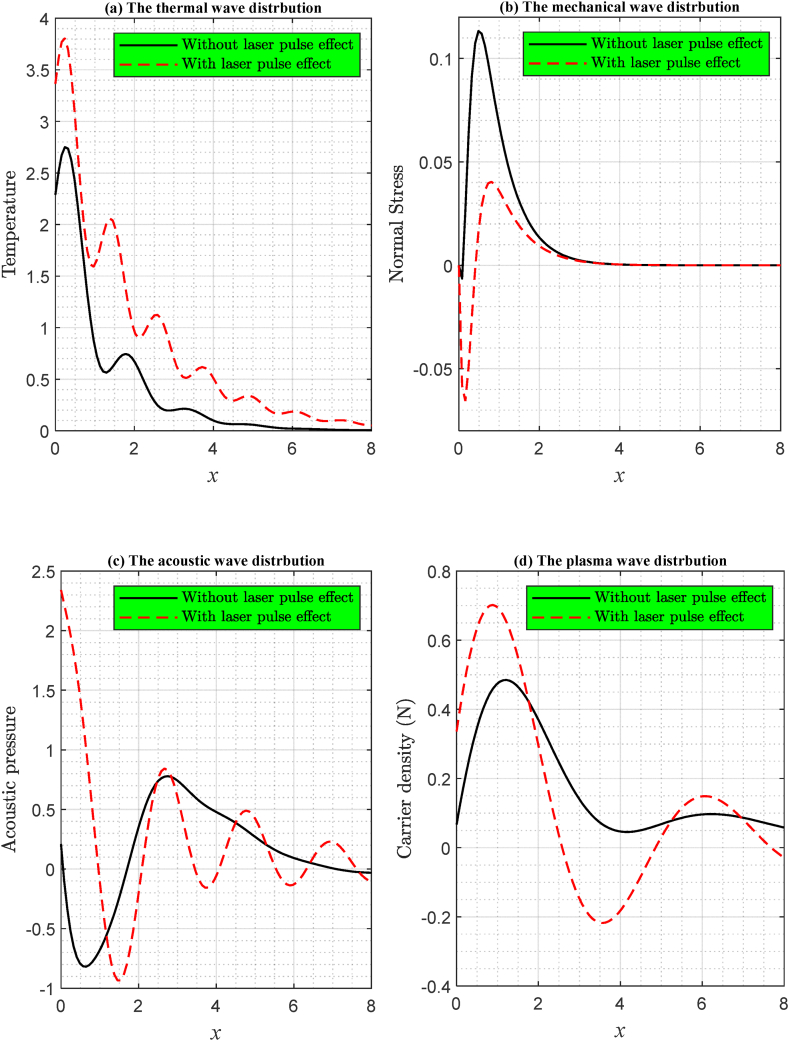
The two-dimensional graph of the main physical fields with and without laser impact with ramp-type heating parameter at $t_{0}=0.02$ according to LS model.

Fig. 3 ((a), (b), (c), (d)) demonstrate the alterations in the primary fields within this phenomenon, contingent upon diverse laser pulse power intensities against a horizontal distance. Following the LS model, the numerical simulation is executed during thermoelastic and electronic deformations over a brief duration under the influence of the ramp-type heating parameter $t_{0}=0.02$. This category encompasses two instances, the first of which pertains to the absence of the laser pulse effect, while the other is subjected to the influence of the laser pulses. It becomes evident that the thermal impact of the laser pulses significantly alters the wave propagation behavior, thereby prompting an investigation into the influence of laser pulses on this particular set of equations.

## 7 Conclusion

Thermal memory and ramp-type heating affect generalized photo-thermoelasticity in an n-type silicon semiconductor exposed to laser pulses. We studied the interaction between thermo-acoustic, mechanical, and plasma waves during elasto-thermodiffusive photoexcitation. In a one-dimensional framework for electronic diffusion, the new model is compared to photo-thermoelasticity theories that use thermal relaxation periods. Thermal memory greatly impacts wave propagation physical distributions. According to photo-thermoelasticity theory, thermal memory are vital to wave propagation of the primary physical fields. Thermal, mechanical, and optical fields in materials can be greatly affected by these slow-moving thermal waves. In materials subjected to rapid thermal and mechanical loads, the theory better depicts the linked behavior of temperature fluctuations, stress waves, and light-matter interactions by accounting for thermal memory effects. We can better understand and predict complicated processes in sophisticated materials and applications. However, laser pulse strength and ramp-type heating parameter affect all wave propagations. Short-term thermal heating in materials produces elastic (acoustic) waves, which are increasingly employed to examine the material's microstructure, especially in semiconductors. In photo-thermoelasticity theory, acoustic pressure is crucial to wave propagation of primary physical fields. Acoustic pressure-induced stress waves interact with thermal and optical fields in materials exposed to photo-thermal stimuli. The connection changes the speed, amplitude, and distribution of these waves in the material. Thus, acoustic pressure greatly affects the associated mechanical, thermal, and optical fields, enabling more accurate predictions and control in non-destructive testing, material characterization, and advanced sensing technologies. These waves propagate at a few kilometers per second, which is surprising given the widespread use of semiconductors in electronics and medicine. These phenomena are important to research because of their high energy concentration on the device's upper surface and their capacity to directly create, detect, and regulate in photovoltaic cells and photonic devices.

## Ethics approval and consent to participate:

Not relevant here for the current paper.

## Funding

Not applicable.

## Data and code availability

No data was used for the research described in the article.

## Data availability

Current submission does not contain the pool data of the manuscript but the data used in the manuscript will be provided on request.

## Use of AI tools declaration

The authors declare they have not used Artificial Intelligence (AI) tools in the creation of this article.

## CRediT authorship contribution statement

**Hashim M. Alshehri:** Validation, Resources, Investigation. **Khaled Lotfy:** Writing – review & editing, Supervision, Formal analysis, Data curation, Conceptualization.

## Declaration of competing interest

No potential conflict of interest was reported by the author.
